# Pancreatic tumor microenvironmental acidosis and hypoxia transform gold nanorods into cell-penetrant particles for potent radiosensitization

**DOI:** 10.1126/sciadv.abm9729

**Published:** 2022-11-11

**Authors:** Pradipta Ranjan Rauta, Yuri Mackeyev, Keith Sanders, Joseph B.K. Kim, Valeria V. Gonzalez, Yasmin Zahra, Muhammad A. Shohayeb, Belal Abousaida, Geraldine V. Vijay, Okan Tezcan, Paul Derry, Anton V. Liopo, Eugene R. Zubarev, Rickey Carter, Pankaj Singh, Sunil Krishnan

**Affiliations:** ^1^ Department of Radiation Oncology, MD Anderson Cancer Center, Houston, TX, USA.; ^2^ Vivian L. Smith Department of Neurosurgery, UTHealth, Houston, TX, USA.; ^3^ Department of Chemistry, Rice University, Houston, TX, USA.; ^4^ Department of Quantitative Health Sciences, Mayo Clinic, Jacksonville, FL, USA.

## Abstract

Coating nanoparticles with stealth epilayers increases circulation time by evading opsonization, macrophage phagocytosis, and reticuloendothelial sequestration. However, this also reduces internalization by cancer cells upon reaching the tumor. We designed gold nanorods (GNRs) with an epilayer that retains stealth properties in circulation but transforms spontaneously in the acidotic tumor microenvironment to a cell-penetrating particle. We used a customized stoichiometric ratio of l-glutamic acid and l-lysine within an amphiphilic polymer of poly(l-glutamic acid-co-l-lysine), or P(Glu-co-Lys), to effect this transformation in acidotic environments. P(Glu-co-Lys)-GNRs were internalized by cancer cells to facilitate potent in vitro radiosensitization. When administered intravenously in mice, they accumulate in the periphery and core of tumors without any signs of serum biochemical or hematological alterations, normal organ histopathological abnormalities, or overt deterioration in animal health. Furthermore, P(Glu-co-Lys)-GNRs penetrated the tumor microenvironment to accumulate in the hypoxic cores of tumors to potently radiosensitize heterotopic and orthotopic pancreatic cancers in vivo.

## Teaser

Acidotic pancreatic tumor microenvironment transforms a circulating Dr. Jekyll nanorod into Mr. Hyde to boost radiosensitivity.

## INTRODUCTION

Homing systemically administered nanoparticles to tumors relies on either passive targeting via the enhanced permeability and retention effect of leaky tumor vasculature or active targeting via decoration of the nanoparticle with tumor-specific biomolecules that facilitate tumor accumulation (*1*–*3*). In both strategies, the journey undertaken by the nanoparticle is treacherous—it is tagged with opsonins in circulation for efficient capture by macrophages in the bloodstream, it is phagocytosed and cleared by resident phagocytic cells in the reticuloendothelial system (RES) organs like the liver and spleen, it has to contact endothelial fenestrations in immature chaotic tumor vasculature to extravasate into the tumor, and it has to overcome the tumor microenvironmental barriers that harbor therapy-resistant cells thriving in a hostile hypoxic milieu (*4*–*7*). The next generation of nanoparticles will have to overcome not just one but as many of these impediments to tumor access as possible. We have designed a nanoparticle that addresses each of these barriers to tumor entry to achieve profound radiation sensitization of a notorious challenging cancer, pancreatic cancer. First, however, we describe features of an ideal nanoparticle-based drug delivery system.

An ideal nanoparticle that accumulates in tumors upon intravenous administration is characterized by a long circulation time and minimal opsonization, circulating macrophage engulfment, and RES uptake (*8*, *9*). This is usually achieved by decoration of the surface with stealth coatings like polyethylene glycol that is near neutral in charge, biocompatible, nonimmunogenic, and nonantigenic; minimizes formation of a protein corona; and mimics biomembranes. The next step is to extravasate from circulation into tumors via wide pores and fenestrations in the endothelial lining of tumor neovasculature. Particles that are anisotropic and nonspherical have a greater opportunity to travel along the edges of a laminar flow column of blood in a vascular channel rather than in the center, thereby maximizing the chances of contacting endothelial cells and tumbling through wide interendothelial junctions and transendothelial channels (*10*–*12*).

Once in the extracellular space of the tumor, ideally the stealth coating is shed to expose the nanoparticle core that can then traverse the cell membrane of cancer cells and deliver its cargo to the tumor cell. This often requires the nanoparticle reacting to extracellular or intracellular tumor stimuli or external stimuli to transform into more cell-interactive forms for enhanced cellular internalization (*3*). This duality of particles whereby they have stealth cloaks in peripheral circulation but shed these cloaks upon arriving at the tumor serves to improve drug delivery to tumors, maximize therapeutic efficacy, and minimize off-target toxicity (*13*, *14*). A number of these stimuli-responsive particles have been designed in the past (*15*, *16*).

Tumor cellular internalization is greatly influenced by the surface charge of the nanocarrier (*17*, *18*). Positively charged nanocarriers show higher affinity than neutral- or negatively charged nanocarriers toward negatively charged cell membranes for easy internalization (*19*). However, the drawback is that positively charged nanocarriers also readily adsorb negatively charged serum proteins on their surface, get opsonized easily, and are rapidly cleared from circulation, or if they escape RES capture, they are taken up by cell nonspecifically (*20*). In contrast, negatively charged or neutral-charged nanocarriers are more resistant to protein adsorption and have longer circulation times suitable for in vivo applications (*21*, *22*). Therefore, it may be ideal to formulate nanocarriers that have a negative or neutral charge during circulation in blood and then transform into a positively charged form in the tumor extracellular environment.

Among the intrinsic features of the tumor microenvironment is extracellular acidity, which can be used to activate a charge reversal on the surface of nanoparticles (*23*, *24*). There are few reports on the use of biodegradable nanocarriers with tumoral extracellular acidity–activated charge reversal features for enhanced cellular uptake (*24*). Biodegradable synthetic polypeptides have been used in biomedical application such as drug delivery, tissue engineering, diagnostics, and biosensors (*25*, *26*). Poly(l-glutamic acid) and poly(l-lysine) are typical polypeptides that have many side groups that are pH responsive with the competition between protonation and deprotonation of lysine and glutamic acid residues driving the pH responsiveness (*27*–*29*). To date, these strategies have been largely confined to in vitro evaluation or have required complex synthetic strategies that are not facile or lead to nonuniform large peptide coatings (*28*). Considering that the tumoral extracellular environment (pH 6.5 to 7.2) is more acidic than that of blood (pH 7.4), this relative acidosis could be exploited for charge reversal of long-circulating neutral- to negatively charged nanocarriers to positively charged cell-penetrating nanocarriers in the tumor microenvironment.

We chose gold nanoparticles (GNPs) as the prototype nanocarrier because of the converging evidence that, when they are internalized by cancer cells, they sensitize tumors to radiation therapy because of their high atomic number (Z) resulting in greater interaction cross sections with incident photons. Then, in principle, the combination of standard-dose radiation therapy (RT) with radiosensitizing GNPs may enhance radiation therapy efficacy locally within tumors leaving adjacent healthy tissues unharmed (*4*, *6*, *30*–*34*). The high atomic number of gold (Z = 79), the high contrast and high mass energy coefficient relative to soft tissue (*35*–*41*), the simple synthesis of particles ranging in size from 1 to 100 nm for easy passage through leaky tumor vasculature, facile surface modification via thiol linkages to PEG and other functional moieties, and the long and safe use of gold in clinical applications make GNPs attractive conduits for high-Z–dependent radiosensitization (*4*, *31*, *39*). The degree and specificity of GNP-mediated radiation sensitization depend largely on the amount and spatial distribution of GNPs within tumors and the extent of internalization by tumor cells. Although GNPs of different shapes and sizes show differing rates of blood vessel extravasation and metabolic clearance (*31*, *34*, *39*, *42*), the preponderance of data suggests that, in comparison to nanospheres, nanorods offer longer systemic circulation time, higher tumor targeting efficiency, superior intratumoral transport, and longer tumor retention time (*11*, *12*, *43*).

Here, we report on a charge-reversal poly(l-glutamic acid-co-l-lysine) [P(Glu-co-Lys)] nanocarrier triggered by tumor extracellular acidity for enhanced cellular uptake and radiosensitization efficiency of gold nanorods (GNRs) in vitro and in vivo. Such a construct fulfills all of the criteria for reaching tumor cells when administered intravenously; i.e., it evades opsonization in circulation because of its near-neutral charge at blood pH, it contacts endothelial fenestrations in tumor vasculature, it switches charge to enter tumor cells, and it is especially efficient at targeting hypoxic and traditionally radioresistant areas of tumors. When it reaches the end of its journey by being internalized by cancer cells, it serves as potent sensitizers of tumors to radiation therapy.

## RESULTS

### Surface charge of purified P(Glu-co-Lys) peptides increases with acidification

**Fig. 1.**
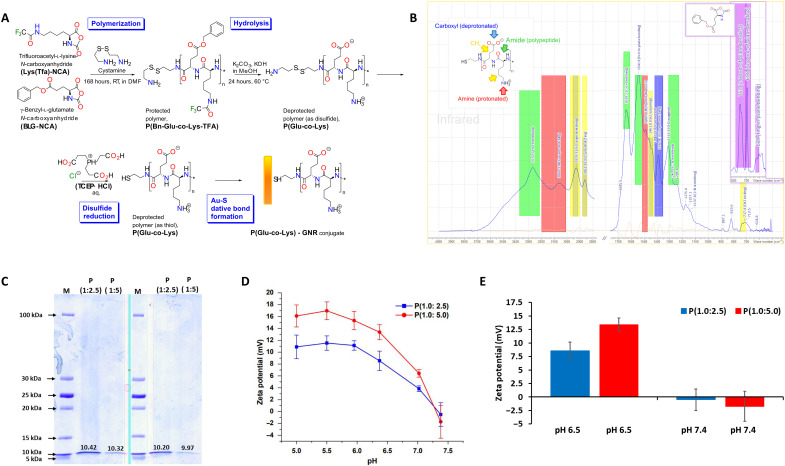
Synthesis and characterization of charge-reversal peptide. (**A**) Schematic of [P(Glu-co-Lys)] synthesis and GNR conjugation. (**B**) Tracking synthesis by FTIR spectrometry analysis. (**C**) The apparent molecular weights (MW) of [P(Glu-co-Lys)1: 2.5] and [P(Glu-co-Lys)1:5] determined by SDS-PAGE gel electrophoresis. (**D**) Relationship between pH and zeta potential (surface charge) of formulated peptides at different pH values: [P(Glu-co-Lys)1:2.5] and [P(Glu-co-Lys)1:5]. (**E**) Change in zeta potential of [P(Glu-co-Lys)1:2.5] and [P(Glu-co-Lys)1:5] between pH 7.4 and 6.5.

The ring-opening copolymerization (ROCOP) synthesis of P(Gly-co-Lys) peptides was performed as described before and is depicted in Fig. 1A. The deprotection progress [removal of benzene and *N*-carboxyanhydride (NCA) groups] was monitored by Fourier transform infrared (FTIR) analysis demonstrating disappearance of peaks (Fig. 1B; ring out-of-plane bending observed as a sharp band at 698 cm^−1^, and C─H out-of-plane bending at 752 cm^−1^, in the BLG-NCA spectrum shown in purple) corresponding to benzyl ester. Also, carboxyanhydride peaks (1772 to 1800 cm^−1^ attributed to cyclic C═O groups and 3331 cm^−1^ attributed to the secondary amine) were not found in the P(Glu-co-Lys) spectrum. The polypeptide α helix amide I bonds around 1640 cm^−1^ and amide II in the range of 1545 cm^−1^ appeared in the IR spectrum. The broad peak around 3270 cm^−1^ due to N–H stretching of amide linkage was also observed (Fig. 1B). FTIR spectra of P(Glu-co-Lys)1:2.5 and P(Glu-co-Lys)1:5 were not notably different. The apparent MW of the synthesized peptides was determined by SDS–polyacrylamide gel electrophoresis (SDS-PAGE) (*44*) to be 10.31 ± 0.15 kDa and 10.14 ± 0.25 kDa for P(Glu-co-Lys)1:2.5 and P(Glu-co-Lys)1:5, respectively (Fig. 1C).

The change of surface charge of P(Glu-co-Lys) at different pH was monitored by observing the peptide ζ-potential (Fig. 1D). With decreasing solution pH, the ζ-potential values of all P(Glu-co-Lys) formulations were observed to increase in response to deprotonation of the copolymer. The Glu/Lys molar ratio strongly influenced the transition pH at which charge reversal occurred. By optimizing the l-glutamic acid/l-lysine ratio, the surface charge of P(Glu-co-Lys) was titrated such that the transition from negative to positive charge occurred at pH values below 7.4 (blood pH; Fig. 1E). Two optimized ratios, P(Glu-co-Lys)1:2.5 and P(Glu-co-Lys)1:5, were chosen for subsequent experiments because of the charge reversal seen within the pH range of 7.4 to 6.5.

### Conjugation of peptides to GNRs durably preserves the charge-reversal properties

**Fig. 2.**
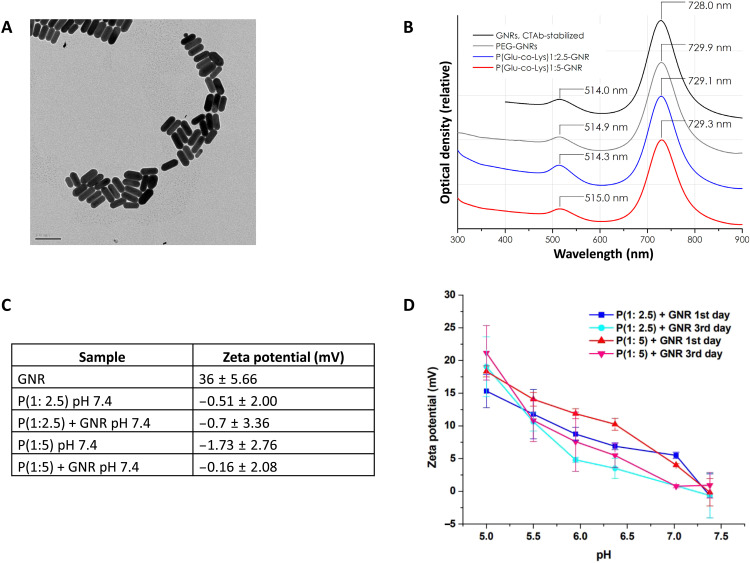
Physicochemical characterization of nanoparticles. (**A**) Transmission electron image GNRs; scale bar, 100 nm. (**B**) UV-vis absorption spectra of GNRs after PEGylation and peptide conjugation showing slight red shift of spectra. (**C**) Table showing change in zeta potential with each step of functionalization. (**D**) Zeta potential of P(Glu-co-Lys)-GNRs as a function of pH and time demonstrating charge reversal and stability.

GNRs synthesized by the seed-mediated growth method were characterized by transmission electron microscopy (TEM; Fig. 2A) and noted to have an average diameter of 25 nm and length of 75 nm. The characteristic bimodal ultraviolet-visible (UV-vis) absorption spectrum experienced a slight red shift of the second peak upon PEGylation or peptide conjugation; the peak wavelength of hexadecyltrimethylammonium bromide (CTAB)–coated GNRs of 728 nm shifted to 729 to 730 nm for peptide-coated GNRs (Fig. 2B). Also noted was the change in ζ-potential after such modification (Fig. 2C). Last, the change of surface charge with pH change was again observed at days 1 and 3 (Fig. 2D). GNR conjugation did not significantly alter the charge reversal pattern of P(Glu-co-Lys) at varying pH values. The P(Glu-co-Lys)-GNRs remained stable in solution for 72 hours after synthesis and retained their charge reversal properties during this period as well.

### Extracellular acidosis increases tumor but not normal cellular uptake of P(Glu-co-Lys)-GNRs

**Fig. 3.**
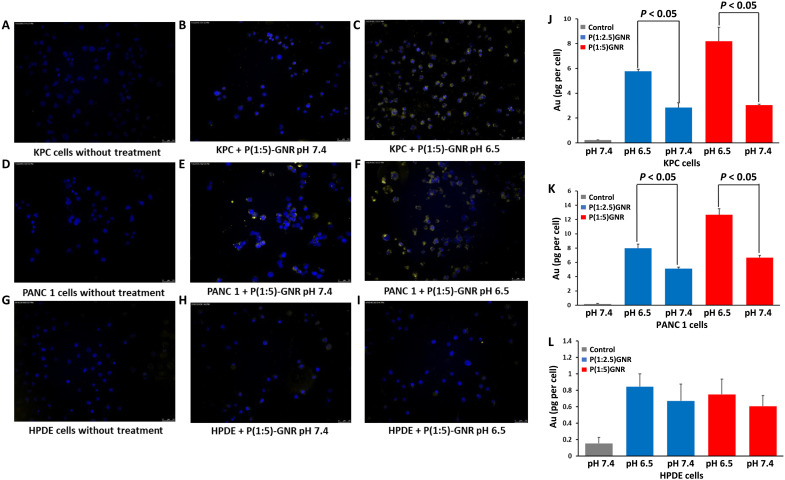
Qualitative and quantitative uptake of nanoparticles in cells. In vitro dark-field and fluorescence overlay images of KPC (**A** to **C**), PANC 1 (**D** to **F**), and HPDE (**G** to **I**) cells incubated with media, P(Glu-co-Lys)1:5-GNR at pH 7.4 and 6.5. Gold uptake was significantly higher in KPC and PANC 1 pancreatic cancer cells at pH 6.5 compared to pH 7.4 for both P(Glu-co-Lys)1:2.5-GNR and P(Glu-co-Lys)1:5-GNR. However, no observable increase in gold uptake was observed in case of HPDE cells. In vitro quantitative uptake of gold measured by ICP-MS analysis of KPC (**J**), PANC 1 (**K**), and HPDE (**L**) cells incubated with media alone (control), P(Glu-co-Lys)1:2.5-GNR, and P(Glu-co-Lys)1:5-GNR at pH 7.4 and 6.5. Gold uptake was significantly higher in KPC and PANC 1 pancreatic cancer cells at pH 6.5 compared to pH 7.4 for both P(Glu-co-Lys)1:2.5-GNR and P(Glu-co-Lys)1:5-GNR. However, no significant increase in gold uptake was observed in case of HPDE cells. Error bars = SEM.

Qualitative analysis of GNR uptake using dark-field and fluorescence microscopy demonstrated superior uptake of P(Glu-co-Lys)-GNR at pH 6.5 compared with pH 7.4 in pancreatic cancer cells (KPC and PANC 1; Fig. 3, A to F). However, there were no such differences observed between pH 7.4 and 6.5 in normal cells [human pancreatic ductal epithelial cells (HPDE); Fig. 3, G to I]. Quantitative measurements of elemental gold concentration within cells by inductively coupled plasma mass spectrometry (ICP-MS) further confirmed the above observation as both P(Glu-co-Lys)1:2.5-GNR– and P(Glu-co-Lys)1:5–treated KPC and PANC 1 cancer cells showed significantly higher cellular uptake at pH 6.5 compared to pH 7.4. However, HPDE cells had low cellular uptake with no observable difference between pH 7.4 and pH 6.5. These results suggest that charge reversal facilitates efficient internalization of P(Glu-co-Lys)1:2.5-GNR and P(Glu-co-Lys)1:5-GNR. We chose P(Glu-co-Lys)1:5-GNR for further experiments based on its better charge-reversal pattern and higher cellular uptake than P(Glu-co-Lys)1:2.5-GNR (Fig. 3, J to L). In an effort to understand drivers of internalization, we blocked microtubule polymerization, clathrin-mediated endocytosis, caveolae-dependent endocytosis, and dynamin with nocodazole, chlorpromazine, nystatin, and dynasore, respectively, and observed that the increased uptake of KPC cells treated with P(Glu-co-Lys)1:5-GNR compared to PEGylated GNR (PEG-GNR) can be reversed by nocodazole, chlorpromazine, and nystatin but not dynasore, suggesting that active transport across the cell membrane also contributes to internalization by cancer cells in an acidotic environment (fig. S1). While caveolin is up-regulated during neoplastic transformation and overexpressed in advanced stages of cancer, the role of clathrin and caveolin in tumor progression remains unclear. In vivo demonstration of relatively low normal tissue uptake (see below) seems, however, to corroborate what we see in vitro.

### Cellular internalization of P(Glu-co-Lys)-GNRs under acidotic or hypoxic conditions enhances in vitro radiosensitivity

**Fig. 4.**
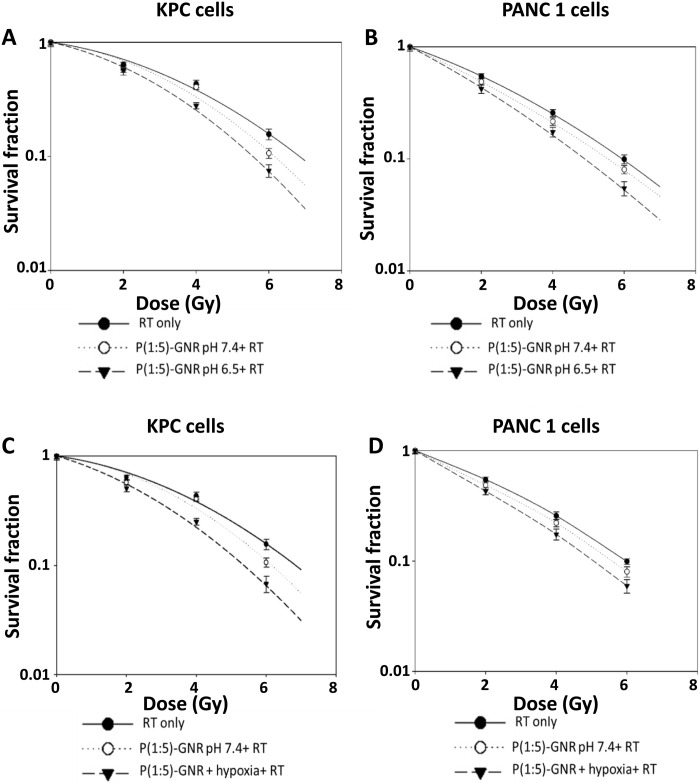
Radiosensitization of pancreatic cancer cells in vitro. Clonogenic survival assay of irradiated KPC (**A**) and PANC 1 (**B**) cells treated with P(Glu-co-Lys)1:5-GNR at pH 7.4 or 6.5. P(Glu-co-Lys)1:5-GNR causes greater radiosensitization at pH 6.5 than at pH 7.4 in both cell lines. Clonogenic survival assay of irradiated KPC (**C**) and PANC 1 (**D**) cells treated with P(Glu-co-Lys)1:5-GNR in the presence or absence of chemical hypoxia (induced by CoCl_2_). P(Glu-co-Lys)1:5-GNR causes greater radiosensitization under hypoxic conditions than normoxic conditions in both cell lines.

Radiosensitization of internalized GNRs was analyzed using classical in vitro clonogenic assays in PANC 1 and KPC cells at pH 7.4 and pH 6.5. As seen in the tumor cell survival curves displayed in Fig. 4 (A and B), P(Glu-co-Lys)1:5-GNR showed significantly enhanced radiosensitization at pH 6.5 compared to pH 7.4 in both PANC 1 and KPC cells. DEF_10%_ of P(Glu-co-Lys)1:5-GNR in KPC cells was 1.06 at pH 7.4 and 1.24 at pH 6.5, whereas the corresponding DEF_10%_ of P(Glu-co-Lys)1:5-GNR in PANC 1 cells was 1.05 and 1.1, respectively. Recognizing that intratumoral oxygen gradient contributes to plasticity and heterogeneity of tumors (*45*, *46*), we then extended these observations from extracellular acidosis mimicking the prototypical tumor microenvironment to the hypoxic milieu of tumors that fosters quiescent, treatment-resistant, epithelial-to-mesenchymal transitioning, metastatic clones that portend a poor overall prognosis and drive progression and metastasis. It is also well recognized that tumor hypoxia induces a metabolic shift, causing acidosis in tumor extracellular space (*47*, *48*). When KPC and PANC 1 cells were treated with the chemical hypoxia-mimetic agent, CoCl_2_ (100 μM, 37°C, 5% CO_2_ for 24 hours), instead of adjusting the extracellular pH of the medium, potent radiosensitization was observed with P(Glu-co-Lys)1:5-GNR in both PANC 1 and KPC cells compared to no treatment with CoCl_2_ (Fig. 4, C and D).

### In vivo biodistribution demonstrates excellent tumor uptake of P(Glu-co-Lys)1:5-GNRs

**Fig. 5.**
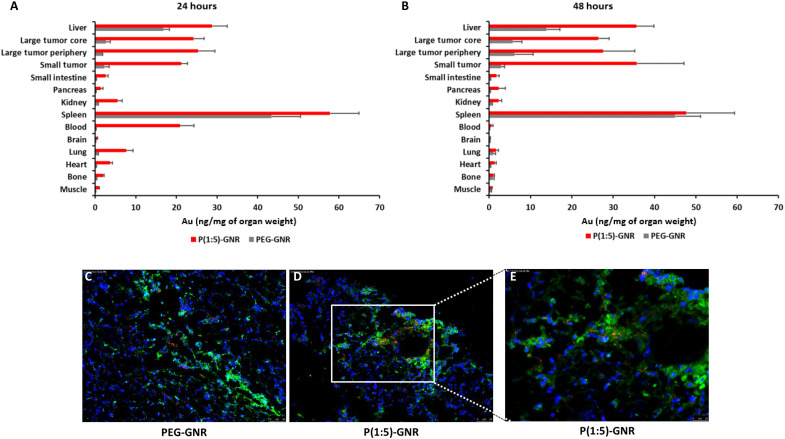
In vivo biodistribution of nanoparticles in normal organs and tumors. Biodistribution analysis by ICP-MS of elemental gold concentration in tumor and normal tissue samples (**A**) 24 hours and (**B**) 48 hours after intravenous administration of P(Glu-co-Lys)1:5-GNR in vivo. Representative 10× magnification overlay images of dark-field and fluorescence microscopy of tumors from (**C**) PEG-GNR– and (**D**) P(Glu-co-Lys)1:5-GNR–treated mice. (**E**) A higher-magnification (20×) zoomed-in image of (D) is also shown. Green fluorescence represents hypoxia stained by pimonidazole, blue fluorescence represents cellular nuclei, and red represents pseudo-colored dark-field images of GNRs.

The in vivo biodistribution study deliberately included large and small tumors such that greater hypoxia, especially within the core of the tumor, could be analyzed by dissecting the large tumors into a central (hypoxic) compartment and a peripheral (nonhypoxic) compartment. As with most GNPs in this size regime, the greatest normal organ concentration of elemental gold at 24 and 48 hours after injection was in the liver and spleen that harbor hepatic stellate and Kupffer cells and splenic macrophages, respectively (Fig. 5, A and B) (*49*–*51*). ICP-MS analysis also revealed significant accumulation of P(Glu-co-Lys)1:5-GNR in both small and large tumors, with comparable accumulation within the periphery and the (hypoxic) core of the large tumors. To generate a more comprehensive biodistribution and kinetics analysis of PEG-GNRs compared to P(Glu-co-Lys)1:5-GNR, elemental analysis of gold content in normal organs, blood, and tumor was performed at multiple longitudinal time points and is presented in the Supplementary Materials (fig. S2).

### Ex vivo analysis of tumor sections demonstrates excellent colocalization between GNRs and areas of tumor hypoxia

While tumor uptake was high in both small and large tumors on ICP-MS analysis, this does not provide information about the spatial distribution of P(Glu-co-Lys)1:5-GNRs, especially in relation to areas of hypoxia that are traditionally the hardest to get nanoparticles to and, therefore, the least likely to exhibit amplified radiosensitization. As seen in Fig. 5 (C to E), when tumor tissue sections were analyzed by superimposing immunofluorescence images of hypoxic areas identified by pimonidazole staining (green) with the location of GNRs identified by dark-field imaging (red), preferential accumulation of GNRs within hypoxic areas was seen in the P(Glu-co-Lys)1:5-GNR–treated group compared to the PEG-GNR–treated group. Blue fluorescence represents 4′,6-diamidino-2-phenylindole (DAPI)–stained cellular nuclei. While rare GNRs were seen in hypoxic areas in the PEG-GNR–treated tumors, considerably more GNRs were spotted in hypoxic areas in the P(Glu-co-Lys)1:5-GNR–treated tumors. This suggests that, probably as a consequence of the greater acidosis in hypoxic areas, there was greater penetration of GNRs into the hypoxic core of tumors when decorated with P(Glu-co-Lys)1:5 than with PEG alone. GNRs reaching these traditionally radiation-resistant cells could significantly increase their response to radiation and thereby radiocurability of cancers. Furthermore, ex vivo analysis of tumors extracted after intravenous administration of rhodamine-lectin and Hoechst 33324 immediately before euthanasia was carried out. Overlaid fluorescence and dark-field images demonstrate that P(Glu-co-Lys)1:5-GNRs and PEG-GNRs penetrate orthotopic pancreatic cancer parenchyma well past tumor vasculature, with the former possibly penetrating farther than the latter (fig. S3).

### P(Glu-co-Lys)1:5-GNR treatment before irradiation resulted in potent tumor regrowth delay

**Fig. 6.**
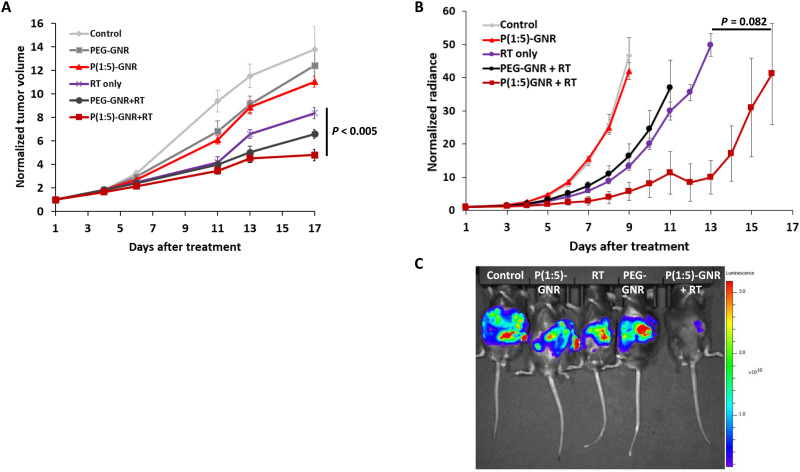
Radiosensitization of pancreatic cancers in vivo. (**A**) In vivo tumor regrowth delay curves of C57BL/6 mice harboring subcutaneous KPC tumors and treated with P(Glu-co-Lys)-GNRs, PEG-GNRs, and/or radiation. The greatest regression in tumor regrowth was observed in the P(Glu-co-Lys)-GNRs + RT group. (**B**) In vivo tumor growth delay curves of C57BL/6 mice harboring orthotopic KPC tumors and treated with P(Glu-co-Lys)-GNRs, PEG-GNRs, and/or radiation. The greatest regression in tumor regrowth was observed in the P(Glu-co-Lys)-GNRs + RT group. (**C**) A representative bioluminescence image of mice from each of the treatment groups on day 11.

Next, we examined the radiosensitization potential of P(Glu-co-Lys)1:5-GNR in a classical in vivo tumor regrowth delay assay. Male C57BL/6 mice harboring subcutaneous KPC tumors (that recapitulate the desmoplasia with exuberant stroma seen in human pancreatic cancers) were randomized into six groups [untreated control, PEG-GNR alone, P(Glu-co-Lys)1:5-GNR alone, RT alone, PEG-GNR + RT, and P(Glu-co-Lys)1:5-GNR + RT]. Animals in the RT groups received a single dose of 8-Gy radiation using a 250-kVp orthovoltage irradiator (Phillips 250) 24 hours after intravenous administration of GNRs (injected gold ~1.42 μg/g of animal body weight). The tumor volume plot (Fig. 6A) demonstrated the greatest delay in tumor regrowth in the P(Glu-co-Lys)1:5-GNR + RT group. On comparing the slopes of the log relative tumor volume versus days curves, P(Glu-co-Lys)1:5-GNR + RT treatment was considerably more effective than RT only with a profound decrease (*P* < 0.005) in the rate of regrowth after day 13 of treatment compared to those treated with RT alone. Mice treated with P(Glu-co-Lys)1:5-GNR + RT also had far smaller normalized tumor volumes compared to RT alone by day 17 of the study (4.81 versus 13.80; Fig. 6B). Similarly, there was a significant decrease (*P* < 0.005) in the rate of regrowth after 13 days of the P(Glu-co-Lys)1:5-GNR + RT–treated tumors compared to the PEG-GNR + RT–treated tumors. As expected, mice treated with PEG-GNR alone or P(Glu-co-Lys)1:5-GNR alone had no significant difference in average normalized tumor volume when compared with each other or with control mice (without RT). A random intercept mixed model treating days after treatment and treatment as fixed, categorical effects resulted in a significant treatment by study day interaction term (*P* < 0.0001). In post hoc comparisons at day 17, P(Glu-co-Lys)1:5-GNR + RT was statistically lower than the other five experimental conditions (*P* < 0.0016 for each, unadjusted *P* value). Individual *P* values for comparison of P(Glu-co-Lys)1:5-GNR + RT with control, PEG-GNR, P(Glu-co-Lys)1:5-GNR alone, RT alone, and PEG-GNR + RT were <0.0001, <0.0001, <0.0001, <0.0001, and 0.0016, respectively.

We then performed an independent experiment in mice harboring orthotopic luciferase-transfected KPC tumors randomized to five groups [untreated control, P(Glu-co-Lys)1:5-GNR alone, RT alone, PEG-GNR + RT, and P(Glu-co-Lys)1:5-GNR + RT]. Animals in the RT groups received a single dose of 8-Gy radiation using a stereotactic irradiator (XRAD SMART) 24 hours after intravenous administration of GNRs (injected gold ~0.7 μg/g of animal body weight). The tumor radiance plot (Fig. 6B) demonstrated the greatest delay in tumor regrowth in the P(Glu-co-Lys)1:5-GNR + RT group. Animals were euthanized after significant tumor burden. Representative bioluminescence images of mice in each group on day 11 are shown in Fig. 6C. On day 7, all animals were still alive. Kruskal-Wallis testing showed overall group differences in tumor size (*P* = 0.0004) with P(Glu-co-Lys)1:5-GNR + RT having a lower tumor burden than the other four groups (*P* < 0.03 except for the RT alone group, which did not achieve statistical significance at *P* = 0.082). These results demonstrate that P(Glu-co-Lys)1:5-GNRs radiosensitize subcutaneous and orthotopic pancreatic cancers even when they harbor large areas of hypoxia within their desmoplastic stroma.

### P(Glu-co-Lys)1:5-GNR treatment is well tolerated with no appreciable toxicity

**Fig. 7.**
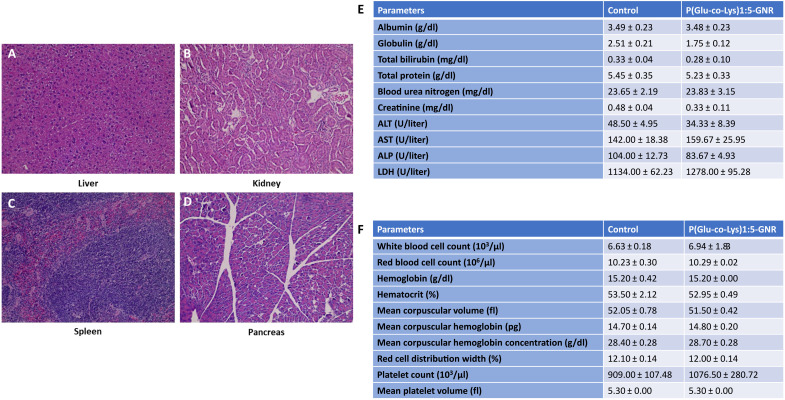
Toxicologic assessment of nanoparticles in vivo. Representative histopathological images (H&E staining) of (**A**) liver, (**B**) kidney, (**C**) spleen, and (**D**) pancreas sections after exposure to P(Glu-co-Lys)1:5-GNR. The light micrographs showed no abnormalities detected in organ sections following exposure to P(Glu-co-Lys)-GNR. Tables representing (**E**) biochemical and (**F**) hematological parameters in blood 24 hours after administration of phosphate-buffered saline or peptide-GNRs to mice.

Last, we assessed the toxicity profile of P(Glu-co-Lys)-GNRs by histopathological assessment of normal organs, primarily focusing on the liver, kidney, spleen, and pancreas. As seen in Fig. 7 (A to D), no appreciable inflammatory, infectious, significant degenerative, or necrotizing lesions were observed in these organs, suggesting that P(Glu-co-Lys)1:5-GNR exposure does not result in any discernable tissue damage or abnormalities following intravenous administration and may be safe and tolerable for clinical use.

In addition to analysis of histopathological changes for biocompatibility and safety of P(Glu-co-Lys)1:5-GNRs, we also analyzed serum chemistries focusing on liver function [alanine aminotransferase (ALT), aspartate aminotransferase (AST), alkaline phosphatase (ALP), lactate dehydrogenase (LDH), bilirubin, albumin, globulin, and total protein], kidney function [blood urea nitrogen (BUN) and creatinine], and hematological function [white blood cell (WBC), red blood cell (RBC), platelet (PLT), and hemoglobin (Hb)]. As seen in the tables in Fig. 7 (E and F), there was no significant difference between mice treated with control [phosphate-buffered saline (PBS)] and P(Glu-co-Lys)1:5-GNRs across all tested biochemical and hematological parameters.

## DISCUSSION

In this study, we demonstrate that reversal of surface charge on slightly negatively charged GNRs coated with a custom amphiphilic polymeric peptide triggered by tumor extracellular acidosis and/or tumor microenvironmental hypoxia facilitates internalization of the GNR and potently sensitizes pancreatic cancer cells in vitro and pancreatic cancers in vivo to radiation therapy. By custom-formulating P(Glu-co-Lys)-GNRs, we address each of the attributes of an ideal drug delivery system: (i) increased circulation time via decoration with near-neutral or slightly negatively charged epilayer that evades opsonization, RES capture, and rapid clearance from the circulation; (ii) anisotropic nonspherical shape of the nanorod that allows greater extravasation and passive accumulation in tumors; (iii) charge reversal in the tumor microenvironment that transforms this construct into a cell-penetrant construct only within the tumor microenvironment where the pH is lower and not in normal organs, thereby reducing off-target toxicity; (iv) remarkable uptake within the hypoxic cores of desmoplastic tumors that are usually impenetrable and harbor the most quiescent, treatment-resistant, epithelial-to-mesenchymal transitioning, and metastasis-prone cells of heterogeneous tumors; and (v) clear therapeutic benefit as a radiosensitizer based on internalization of a high-Z element into tumor cells. Rather than individual innovations that only address isolated hurdles faced by nanoparticles on the treacherous journey from intravenous injection site to the tumor, our nanoconstruct overcomes multiple barriers to achieve a clinically relevant outcome, i.e., radiosensitization of a recalcitrant therapy-resistant tumor. Given that the formulation is not custom-tailored to pancreatic cancer, as with active targeted to cell surface receptors found only in pancreatic cancer, we envision the P(Glu-co-Lys)1:5-GNR being a class solution for a wide range of tumors independent of histology or genetic makeup.

Although charge reversal is an oft-invoked attribute of stimulus-responsive particles, our approach is especially noteworthy because of its simplicity, scalability, ability to address multiple barriers faced by drug delivery systems, lack of toxicity, and demonstrable improvement in therapeutic efficacy both in vitro and in vivo. We are especially excited about the possibility that P(Glu-co-Lys)-GNRs might overcome hypoxic radioresistance because this is a rarely addressed source of therapeutic failure despite being among the most potent drivers of recurrence and relapse. Hypoxic cells are three times as resistant to radiation as normoxic cells and few radiation treatment strategies have proven effective in countering this source of resistance. Approaches used to date include the use of nitroimidazoles as hypoxic cell sensitizers (*52*), tirapazamine as a bioreductive hypoxic cytotoxin (*53*), and carbon ion radiation as a form of high linear energy transfer treatment that does not require oxygen for “fixation” of DNA damage that it causes “directly” as opposed to x-ray radiation that causes both direct and indirect (predominantly) DNA damage via oxygen free radicals that “fix” the DNA damage (*54*). However, the clinical results with the use of nitroimidazoles have been inconclusive (*52*), that with the use of tirapazamine have suffered from lack of clear efficacy and considerable toxicity (*55*), and that with carbon ion therapy have been inadequately studied because of expense, nonavailability, and lack of good correlative studies (*56*). Within this landscape of sparse treatment options targeting hypoxia for radiosensitization (*57*, *58*), delivery of a concomitant boost dose of radiation to hypoxic foci within tumors that have internalized GNR might be a unique and attractive therapeutic option.

Together, our results suggest that a customized P(Glu-co-Lys)-GNR construct can be designed to overcome multiple barriers to efficient systemic delivery of nanoparticles to tumors and could serve as a turn-key class solution to GNP-mediated radiosensitization of a variety of tumors independent of histology or genetic makeup, thereby easing its path to scale-up manufacturing and regulatory approval. Our results also suggest that desmoplastic pancreatic cancers harboring hypoxic cells that are radiation resistant and biologically aggressive can be sensitized to radiation using systemically administered stealth polymer peptide-coated GNRs that transform to cell-penetrant particles in regions of tumor microenvironmental acidosis and/or hypoxia, a major driver of therapeutic resistance and biological aggressiveness of tumors.

## METHODS

### Synthesis of P(Glu-co-Lys) peptide

The “normal amine mechanism” ring opening polymerization (*59*, *60*) of α-(amino acid)–*N*-carboxyanhydride (NCA), a widely used nucleophile-initiated way of fabricating biomimetic α-peptide materials with precisely engineered properties (*61*–*64*), was used to synthesize a series of polypeptides of glutamic acid and lysine, i.e., P(Glu-co-Lys). Cystamine (Sigma-Aldrich Corp, St. Louis, MO) was selected as an initiator for the amine-mediated ROCOP of γ-benzyl-l-glutamate NCA (BLG-NCA, TRC Canada, Toronto, ON) and *N*-trifluoroacetyl-l-lysine NCA [Lys(Tfa)-NCA, Biosynth International Inc., San Diego, CA] and added at a molar ratio of 1% of total NCA.

By changing the molar ratios of BLG-NCA to Lys(Tfa)-NCA, the surface charge of P(Glu-co-Lys) was varied over a range of pH values, with the greatest emphasis on the range between 7.4 and 6.5, the typical gradient between the vascular compartment pH and the tumor extracellular matrix pH. Typically, P(Glu-co-Lys) copolymers were prepared in the following manner. BLG-NCA and Lys(Tfa)-NCA in a molar ratio 1:5 or 1:2.5 were dissolved in anhydrous *N*,*N*-dimethylformamide (DMF). Cystamine in anhydrous DMF was added, the vial was sealed, and the reaction was allowed to proceed for 7 days at 25°C with stirring. A white solid precipitate was obtained after drying under vacuum at room temperature. Water-soluble P(Glu-co-Lys) polymer was obtained after deprotection of an insoluble P(Bn-Glu-co-Lys-TFA) with 0.1 M K_2_CO_3_ and 0.1 M KOH solution in 95% methanol at 60°C for 24 hours. The disulfide group of cystamine was reduced to the thiol by the addition of 0.1 M aqueous tris(2-carboxyethyl)phosphine hydrochloride (TCEP·HCl, Sigma-Aldrich).

Further removal of salts and oligomeric proteins with low molecular weight (MW) was performed by dialysis using Amicon Ultra-15 10,000 molecular weight cut-off (MWCO) centrifugal filter units with regenerated cellulose membrane; the final product was dried in vacuum. The deprotection progress (removal of benzyl and trifluoroacetate groups) was monitored by FTIR analysis. SDS-PAGE was used for the characterization of synthesized peptides.

### Synthesis of GNRs

Seed-mediated synthesis of GNRs was performed according to methods described elsewhere (*21*, *65*–*67*). Briefly, the gold seed particles were prepared by adding 250 μl of 0.01 M aqueous solution of hydrogen tetrachloroaurate trihydrate (HAuCl_4_·3H_2_O, >49% metals basis, Strem, Newburyport, MA) to 7.5 ml of a 0.1 M CTAB (>99%; Sigma-Aldrich, St. Louis, MO) solution in a 15-ml test tube. Afterward, 600 μl of an ice-cold aqueous 0.01 M sodium borohydride (NaBH_4_, 99%; Acros Organics, Carlsbad, CA) solution was added all at once. This seed solution was used within 2 to 4 hours after its preparation.

The growth solution was prepared by sequentially combining 4.75 ml of 0.1 M CTAB, 200 μl of 0.01 M HAuCl_4_, and 30 μl of 0.01 M silver nitrate (AgNO_3_, Fisher Chemical, Waltham, MA) solutions and gently mixed by inverting the vial. The solution at this stage had a bright orange color. Afterward, the solution was thermostated for 24 hours at 30°C. The CTAB-GNRs were centrifuged at 1000*g* for 10 min to separate aggregates before surface functionalization with mPEG-SH. The pellet was removed and only the supernatant fraction was retained.

### Functionalization of GNRs

PEG-GNRs were prepared using previously synthesized CTAB-coated GNRs (*21*, *68*). The GNRs were centrifuged at 14,000*g* for 10 min and resuspended in 9 ml of milli-Q (18.0 megohms) water to obtain the GNR suspension with optical density ~ 1.0. Next, 0.1 ml of 2 mM potassium carbonate (K_2_CO_3_, 99.99%) was added to 1 ml of aqueous GNR solution and 0.1 ml of 0.1 mM mPEG-SH-5 kDa (Laysan Bio Inc., Arab, AL). The resulting mixture was kept on a rocking platform at room temperature overnight. Excess mPEG-SH was removed from solution by two rounds of centrifugation (12,000*g*, 10 min) and resuspended in deionized (DI) water to maintain a final GNR concentration of 0.5 nM (OD ~2.0). UV-vis extinction spectra were measured by spectrophotometer.

GNRs were conjugated to [P(Glu-co-Lys)] through Au-S dative interaction with thiol (-SH) groups without affecting the carboxyl:amino ratio responsible for charge reversal. This was accomplished by combining 1 ml of suspension of CTAB-stabilized GNRs with OD = 2 with 1 ml of protein solution, prepared with 0.5 mg of P(Glu-co-Lys)1:5 or P(Glu-co-Lys)1:2.5 in 0.05 M sodium phosphate buffer (pH 6.9). The peptide-coated GNR suspensions were dialyzed using 50-kDa biotech-grade cellulose ester membranes (Spectra/Por G235034) in 18-megohm DI water (Millipore) until phosphate and CTAB were completely removed, which was traced by measuring the electrical conductivity.

PEG-GNRs were prepared by combining 1 ml of suspension of CTAB-stabilized GNRs with OD = 2 with 1 ml of PEG solution, prepared with 0.5 mg of 10-kDa HS-PEG-OMe in 0.05 M sodium phosphate buffer (pH 6.9), and purified as above.

### Characterization of GNRs

TEM was performed with a JEOL 1230 Field Emission Gun instrument. The prepared peptides and GNR-peptide conjugates in 10-mm quartz cuvettes in DI water were characterized by UV-vis absorbance spectrometry on a Varian CARY-100 BIO spectrophotometer over a range of 200 to 900 nm, with DI water used as the reference.

FTIR spectra were acquired with a Thermo Nicolet 6700 spectrometer (Thermo Fisher Scientific, Waltham, MA) equipped with a Smart iTR accessory for ZnSe crystal ATR sampling (PIKE Technologies, WI) using an N_2_-purged sample chamber. Data were collected between 4000 and 600 cm^−1^, 256 scans, 2 cm^−1^ spectra resolution at room temperature.

The zeta (ζ)–potential values and average hydrodynamic particle diameters (*d*_h_) of all products were obtained using a ZEN3600 Malvern Zetasizer equipped with a 633-nm laser (Worcestershire, UK) using DTS1070 folded capillary zeta cells. Analyses were performed in DI water with the electrical conductivity adjusted to 500 to 1000 μS/cm by the addition of phosphate buffer with pH 5.1 to 8.0; each sample was measured three to five times at 25°C with a protein concentration of 2 mg/ml, with each measurement composed of an average of 50 runs, and the data were presented as averages with SDs. The GNRs samples were centrifuged and diluted with Milli-Q water before each triplicate measurement. The sample masses were recorded with a Mettler Toledo UMX2 microbalance (Mettler Toledo GmbH, Switzerland) with 0.1-μg precision until no change in mass was observed within the sensitivity limits of the balance.

### Quantification of gold concentration

For UV-vis spectra collection, the same GNR stock was diluted 1:10 in Milli-Q water (18 megohms) to 1 ml and centrifuged at 12,000*g* for 15 min. The concentration of gold in the final GNR suspension was determined by dissolving 10 μl with ~0.1 ml of hot aqua regia (1:3 HNO_3_:HCl), diluted with 2% HCl to 10 ml, and performing an ICP–optical emission spectrometry (ICP-OES) analysis using Au^3+^ standards, prepared by serial dilution of a reference standard (999 mg·liter^−1^, Fisher) (*68*). GNR size distribution was obtained by analysis of a selection of 200 nanorods from TEM images using ImageJ (National Institutes of Health). On the basis of the maximum extinction at the surface plasmon resonance wavelength (optical absorption at 728 nm) in the UV-vis spectrum, the gold content in a sample (determined by ICP-OES), and the sample volume, the molar extinction coefficient (ε) of synthesized GNRs was calculated to be 2.6 × 10^10^ M^−1^ cm^−1^. This value was used for the adjustment of working concentrations.

Elemental gold content in in vitro and in vivo samples treated with PEG-GNR and P(Glu-co-Lys)-GNR conjugates was quantified by ICP-MS after sample preparation by the hot plate dissolution technique using concentrated acids. Briefly, the tissue samples were freeze-dried for 48 hours, then carefully weighed, and solubilized by treatment with nitric (Fisher A467-1) and hydrochloric (Fisher A466-1) acids under a fume hood. Solubilization of each was performed as follows. First, 2.5 ml of nitric acid was added to the sample and allowed to stand for 24 hours at room temperature. Then, 0.5 ml of hydrochloric acid was added, and the vial was closed by the screw cap with a PTFE preslit liner and kept on top of a hot plate at 90°C for 48 to 240 hours, until the liquid inside became completely clear. The vial was cooled to room temperature, the cap was carefully opened, and the open-top vial was allowed to stand on the hot plate at 120°C until the volume content reduced to approximately 0.5 ml; then, the residue was diluted with aqua regia (1% nitric acid and 3% hydrochloric acid in DI water), and the final volume was adjusted to 10 ml. The solutions were filtered using 0.2-μm GMF syringe filters (Whatman PLC, Florham Park, NJ) into 15-ml PP Eppendorf tubes and subjected to ICP-MS analysis on an Agilent 7900 ICP-MS quadrupole system to estimate the amount of elemental gold. Gold concentrations were calculated from the regression equation built with a set of standards with concentrations of 1 × 10^−9^ to 1 × 10^−4^ g liter^−1^ prepared by diluting the Au primary standard (999 mg liter^−1^; Fisher). A 0.10 μg liter^−1^ Rb^+^ internal standard was added to the blank, all calibration standards, and samples. The Rb^+^ standard was prepared by diluting the Rb^+^ primary standard (1000 mg liter^−1^; Millipore Sigma) in a PP volumetric flask, and diluting to the desired volume with DI water. For the more extensive biodistribution analysis over multiple time points and the endocytosis inhibition experiments (see below) in the Supplementary Materials, we used identical methodology but ran samples on a PerkinElmer NexION 2000 ICP-MS instead of the Agilent 7900.

### In vitro cellular uptake

To quantitatively measure cellular internalization of P(Glu-co-Lys)-GNR at different pH values (7.4 and 6.5), PANC 1 human pancreatic cancer cells (obtained from American Type Culture Collection, authenticated by STR-based DNA profiling) and KPC murine pancreatic cancer cells derived from the transgenic KrasLSL.G12D/+; p53R172H/+; PdxCretg/+ C57BL/6 mouse (a gift from T. Arumugam at MD Anderson Cancer Center, authenticated by IDEXX STR-based DNA profiling and multiplex polymerase chain reaction to detect both contamination and misidentification of your cell lines) were grown in 60-mm petri dishes (Corning 353004) and incubated with 2 ml of complete media (pH 7.4 and pH 6.5) containing 0.5 OD P(Glu-co-Lys)-GNR for 24 hours. After washing twice with PBS, cells were trypsinized with 0.5 ml of 0.05% trypsin-EDTA (Thermo Fisher Scientific 25300-054), neutralized with 2 ml of complete media, and centrifuged at 400*g* for 5 min. Cells were then resuspended in 1 ml of PBS, transferred to glass scintillation vials (Sigma-Aldrich Z190527), and evaporated at 56°C overnight. Cells were digested in aqua regia, and the elemental gold content in each sample was quantified by ICP-MS against serially diluted Au standards and normalized to cell count. To qualitatively visualize cellular internalization of P(Glu-co-Lys)-GNR, cells were grown in eight-well chamber slides (Thermo Fisher Scientific 154453) and incubated with media, 0.5 OD P(Glu-co-Lys)-GNR for 24 hours, stained with DAPI to visualize the nucleus, and mounted with ProLong Gold Antifade Reagent (Thermo Fisher Scientific P10144). Chamber slides were visualized by fluorescence and dark-field microscopy (Leica DM1000). In addition, normal HPDE cells cultured in keratinocyte serum-free medium (Invitrogen) were treated similarly with 0.5 OD P(Glu-co-Lys)-GNR or 2 OD PEG-GNR for 24 hours and then stained and visualized by fluorescence and dark-field microscopy (Leica DM1000) to compare the uptake in normal pancreatic cells to that in pancreatic cancer cells. For the blocking study with different inhibitors of endocytosis, we quantified elemental gold content in cells by ICP-MS after treatment with 0.5 OD P(Glu-co-Lys)-GNRs and PEG-GNRs for 24 hours in the presence or absence of 2 μM nocodazole (microtubule depolymerizer; 30-min incubation), 100 μM chlorpromazine (clathrin-mediated endocytosis inhibitor; 1-hour incubation), nystatin (50 μg/ml; caveolae-dependent endocytosis inhibitor; 30-min incubation), and 100 μM dynasore (dynamin inhibitor; 1-hour incubation). 

### In vitro clonogenic assay

To assess in vitro radiosensitization, PANC 1 and KPC cells grown in 60-mm petri dishes were incubated with 2 ml of complete media (pH 7.2 and 6.5) containing 0.05 OD P(Glu-co-Lys)-GNR. After 24 hours, cells were washed twice with PBS and exposed to Cu-filtered 250-kVp x-rays (Philips RT-250) at 0, 2, 4, or 6 Gy. The dose rates (in water) for the Cu-filtered 250-kVp x-ray beam were determined by ion chamber measurements following the AAPM TG-61 protocol and found to be 48 and 115 cGy/min for standard irradiation geometries (15 mA, 10 cm by 10 cm field size, and 50-cm source-to-chamber/object distance), respectively. Immediately following x-ray irradiation, cells were counted and reseeded in six-well plates (Corning 3516) and incubated for 10 days before being stained with crystal violet. Plating efficiency was calculated by dividing the number of colonies present in nonirradiated wells by the number of cells seeded. Surviving fraction for each treatment condition was calculated by dividing the number of colonies by the product of the number of cells seeded and the plating efficiency. Dose enhancement factor at 10% surviving fraction (DEF_10%_) was calculated by dividing the radiation dose needed to reduce the surviving fraction to 10% in the radiation only group by the dose needed to reach the same end point in the radiation + GNR groups. In total, two independent clonogenic experiments were performed, each with six replicates. A separate experiment was conducted where KPC and PANC 1 cells were treated with hypoxia-mimetic agent CoCl_2_ (100 μM, 37°C, 5% CO_2_) for 24 hours instead of adjusting the pH of the medium to 6.5 and then the clonogenic assay was performed as discussed above.

### In vivo subcutaneous tumor model

Male C57BL/6 mice 6 to 8 weeks old upon acquisition from The University of Texas MD Anderson Cancer Center’s Experimental Radiation Oncology Mouse Facility were handled following approved Institutional Animal Care and Use Committee (IACUC) protocols at MD Anderson Cancer Center. Roughly 1 × 10^6^ KPC cells were injected into the right thighs of these mice and allowed to grow undisturbed until they reached an average of 5 to 8 mm in diameter before being treated in all in vivo experiments. To create tumors that were less likely to harbor hypoxic cores, half-a-million cells were injected into the left thighs of some mice, as noted below.

### In vivo orthotopic tumor model

Male C57BL/6 mice 6 to 8 weeks old upon acquisition from the Jackson Laboratory (Bar Harbor, ME) were handled following the approved IACUC protocols at Mayo Clinic. After anesthesia with intraperitoneal administration of a ketamine/xylazine mixture and analgesia with subcutaneous carprofen (5 mg/kg), the chest and abdomen were cleaned with betadine solution followed by 70% ethanol, and a gauze sponge with a ~2-cm diameter hole was placed over the left upper quadrant of the abdomen. A small ~1-cm vertical incision through skin and peritoneum was made slightly medial to the splenic silhouette. Using blunt-nose forceps, the spleen and the entire pancreatic body were pulled out of the peritoneal cavity, and a gentle knot was made with a 4-0 polysorb suture around the pancreatic tail ~2 mm from the splenic insertion allowing retraction of the tail while injecting 50 μl of cell suspension containing roughly 1 × 10^6^ KPC cells in Matrigel into the pancreatic body via a 27-G needle. The resulting fluid-filled region in the pancreatic parenchyma was sealed off by tightening the knot to prevent leakage. The externalized pancreas was left untouched to confirm lack of hemorrhage or leakage before being returned to the peritoneal cavity with blunt forceps. The abdominal incision was closed in sequential layers with interrupted 4-0 polysorb sutures, and an additional wound clip (BD427638) and neosporin antibiotic were placed over the incision. Mice were placed on a heat pad and buprenorphine (0.05 mg/kg) was given subcutaneously every 12 hours for 3 days and carprofen (5 mg/kg) was given subcutaneously every day for 3 days. Tumors were allowed to grow undisturbed for a week before initial bioluminescence imaging on an IVIS 200 imaging system (Caliper Life Sciences) on day 8 for randomization. In a separate set of mice with orthotopic tumors, when tumors were less than 1 cm, intravenous injections of 60 μl of rhodamine-lectin (5 mg/ml; RL-1082-5, Vector Laboratories) and 100 μl of Hoechst 33342 (150 mg/ml; ENZ-52401, Enzo Life Sciences) were performed immediately before euthanasia, and the tumor was harvested for immunofluorescence and dark-field imaging at euthanasia.

### Biodistribution study

Six-week-old male C57BL/6 mice (three mice in each treatment group) were injected with ~0.5 × 10^6^ (left thigh) and ~1 × 10^6^ (right thigh) KPC cells to develop small (nonhypoxic) and large (hypoxic) tumors, respectively. Then, the mice (three groups) were intravenously injected with 100 μl of 10 OD PEG-GNRs (control), P(Glu-co-Lys)1:5-GNRs, or P(Glu-co-Lys)1:2.5-GNRs. Then, the elemental gold content of normal organs and tumors harvested 24 and 48 hours after injection was quantified by ICP-MS after lyophilization, complete aqua regia digestion, and filtration. This was repeated in another time course biodistribution study with four mice per treatment group and six time points (30 min, 1 hour, 4 hours, 8 hours, 24 hours, and 48 hours).

### Histopathological assessment of normal organ toxicity

A portion of the liver, kidney, spleen, and pancreas of mice treated with PEG-GNRs, P(Glu-co-Lys)1:5-GNRs, or P(Glu-co-Lys)1:2.5-GNRs was fixed in 10% formalin and embedded in paraffin blocks. These tissues were microtome-sectioned and stained with hematoxylin and eosin. The mounted slides were scored for adverse pathological changes on a Zeiss Axiostar light microscope.

### Hematological and biochemical analysis

Twenty-four hours after treatment with GNRs, blood samples were collected by intracardiac puncture following ketamine/xylazine anesthesia. For hematological analysis, blood was immediately collected in EDTA-coated vials and hematologic toxicity was determined by the use of an automated hematological analyzer. Hematological parameters examined in this study included WBC counts, RBC counts, Hb levels, hematocrit, mean corpuscular volume, mean corpuscular Hb, mean corpuscular Hb concentration, red cell distribution width, PLT count, and mean platelet volume.

For biochemical analysis, serum from the same blood collection was analyzed by an automated analyzer (Hitachi 912 Chemistry Analyzer) to determine liver function as measured by albumin, globulin, total protein, AST, ALT, ALP, total bilirubin, and LDH, and kidney function as measured by BUN, and creatinine.

### Visualizing colocalization of GNRs and areas of hypoxia

In the mice euthanized 24 hours after GNR injection, 2.5 mg of pimonidazole (Hypoxyprobe, Burlington, MA, USA) was administered intravenously 1 hour before euthanasia. Tumors were excised and a portion was embedded in optimal cutting temperature compound for frozen section analysis. The frozen tissues were processed for histological analysis by immunostaining with fluorescein isothiocyanate–anti-pimonidazole immunoglobulin G1 monoclonal antibody. The fluorescence images were then overlaid with the dark-field images to visualize colocalization of GNRs within areas of hypoxia.

### In vivo subcutaneous tumor regrowth delay

Tumor-bearing mice were randomly divided into six groups containing 8 to 10 animals each: control (vehicle treatment without nanoparticles and radiation), radiation alone (RT), P(Glu-co-Lys)1:5-GNR treatment alone, P(Glu-co-Lys)1:5-GNR treatment with radiation [P(Glu-co-Lys)-GNR + RT], PEG-GNR treatment alone (PEG-GNR), and PEG-GNR treatment with radiation (PEG-GNR + RT). Mice in the nanoparticle treatment groups received a single tail-vein injection of P(Glu-co-Lys)-GNR or PEG-GNR (100 μl of 10 OD GNRs). Animals that received RT were anesthetized using an intraperitoneal ketamine and xylazine mixture and then treated with a single dose of 8-Gy radiation using a 250-kVp irradiator (Phillips 250 orthovoltage irradiator) 24 hours after nanoparticle treatment. Digital caliper measurements of the long and short axes of tumor dimension were recorded roughly every 3 days and tumor volume was calculated according to the formula: *v* = *ab*^2^/2, where *v* = tumor volume (mm^3^), *a* = long axis (mm), and *b* = short axis (mm). The mean tumor volume for each group was plotted over time until tumors reached a diameter of 1.5 cm in the long axis, at which point mice were euthanized. Posttreatment tumor volumes were normalized to the starting tumor volume for each animal.

### In vivo orthotopic tumor regrowth delay

Tumor-bearing mice were randomly divided into five groups containing five to six animals each: control (vehicle treatment without nanoparticles and radiation), radiation alone (RT), P(Glu-co-Lys)1:5-GNR treatment alone, P(Glu-co-Lys)1:5-GNR treatment with radiation [P(Glu-co-Lys)-GNR + RT], and PEG-GNR treatment with radiation (PEG-GNR + RT). Mice in the nanoparticle treatment groups received a single tail-vein injection of P(Glu-co-Lys)-GNR or PEG-GNR (100 μl of 10 OD GNRs). Animals that received RT were anesthetized using an intraperitoneal ketamine and xylazine mixture and then treated with a single dose of 8-Gy radiation using a 225-kVp small animal irradiator (XRAD SMART irradiator, PXI Inc.) 24 hours after nanoparticle treatment. Irradiation geometry was based on a preplan using a 360° arc around an isocenter placed at the center of mass of the bioluminescence image. Daily bioluminescence images were obtained 10 min after intraperitoneal injection of 200 μl of freshly prepared IVISbrite D-luciferin potassium salt (15 mg/ml) in PBS. After subtracting background, radiance values in photons/steradian/cm^2^/s over a uniform region of interest centered on the tumor were recorded from daily imaging. Posttreatment radiance values were normalized to the baseline pretreatment radiance values for each animal and plotted over time until mice were euthanized when they became moribund, lost weight, or developed severe ascites.

### Statistical analysis

Each in vitro experiment was conducted in triplicate and data are expressed as means ± SD/SEM. Differences among groups were analyzed by using one-tailed Student’s *t* tests and two-way analysis of variance, as indicated in the captions. A two-tailed *P* value of < 0.05 was considered as statistically significant. For the in vivo biodistribution and tumor growth delay data, the mean values and associated SEM values for each group were calculated and the differences among groups were analyzed using the Mann-Whitney test. Statistical significance was defined as *P* < 0.05. The analysis was performed using SPSS 2.0 software. Specifically, for the tumor growth curve analysis, a random intercept mixed model treating days after treatment and treatment as fixed, categorical effects was generated using R Statistical Software (v4.1.2; R Core Team 2021).

### Ethical conduct of the experiments

All animal experiments were performed in accordance with the guidelines and regulations mentioned in the IACUC protocols at MD Anderson Cancer Center and Mayo Clinic.

## Notes

View/request a protocol for this paper from *Bio-protocol*.
