# Supplementary material for: Pancreatic tumor microenvironmental acidosis and hypoxia transform gold nanorods into cell-penetrant particles for potent radiosensitization
Source: Sci Adv. 2022 Nov 11;8(45):eabm9729. doi: 10.1126/sciadv.abm9729 (PMC9651859; doi:10.1126/sciadv.abm9729)
Supplement: Supplementary file 1 — Figs. S1 to S3 [file sciadv.abm9729_sm.pdf]

Science Advances
AAAS

# Supplementary Materials for

## Pancreatic tumor microenvironmental acidosis and hypoxia transform gold nanorods into cell-penetrant particles for potent radiosensitization

Pradipta Ranjan Rauta *et al.*

Corresponding author: Sunil Krishnan, sunil.krishnan@uth.tmc.edu

*Sci. Adv.* **8**, eabm9729 (2022)
DOI: 10.1126/sciadv.abm9729

**This PDF file includes:**

Figs. S1 to S3

**Supplementary Materials**

**Figure S1**: Inductively coupled plasma – mass spectrometry analysis of gold uptake by KPC cells treated at pH6.5 with inhibitors of endocytosis before treatment with P(Glu-co-Lys)1:5-GNR and PEG-GNR showing inhibition of uptake when cells are treated with nocodazole, chlorpromazine and nystatin which block microtubule polymerization, clathrin-mediated endocytosis, and caveolae-dependent endocytosis, respectively.

**Figure S2**: In vivo biodistribution analysis of C57BL/6 mice bearing subcutaneous KPC tumors and treated with P(Glu-co-Lys)1:5-GNR and PEG-GNR before elemental analysis of gold content in normal organs, blood, and tumors at different time points.

**Figure S3**: Ex vivo analysis of depth of penetration of P(Glu-co-Lys)1:5-GNR and PEG-GNR away from vasculature upon intravenous administration 24 h before harvesting orthotopic KPC tumors from C57BL/6 mice. Mice received intravenous rhodamine-lectin to stain vasculature and Hoechst 33342 to document perfusion; these dyes were visualized by fluorescence microscopy and the nanoparticle clumps were visualized by dark field microscopy.

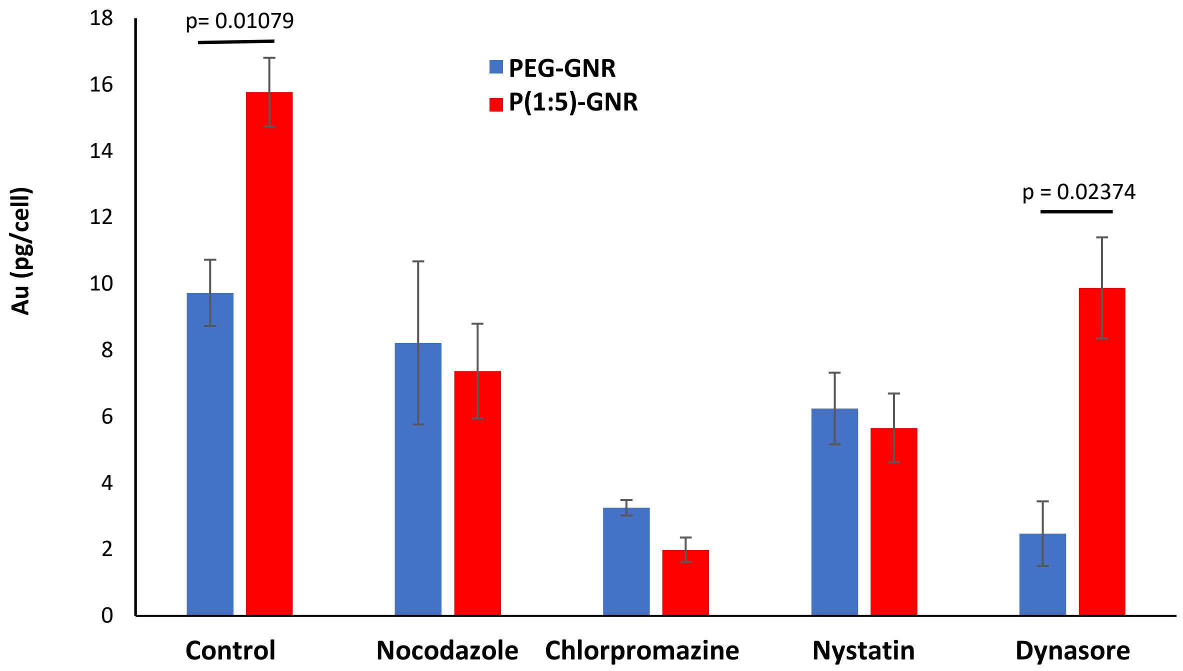
p= 0.01079
PEG-GNR
P(1:5)-GNR
p = 0.02374
Au (pg/cell)
18
16
14
12
10
8
6
4
2
0
Control
Nocodazole
Chlorpromazine
Nystatin
Dynasore

Figure S1.

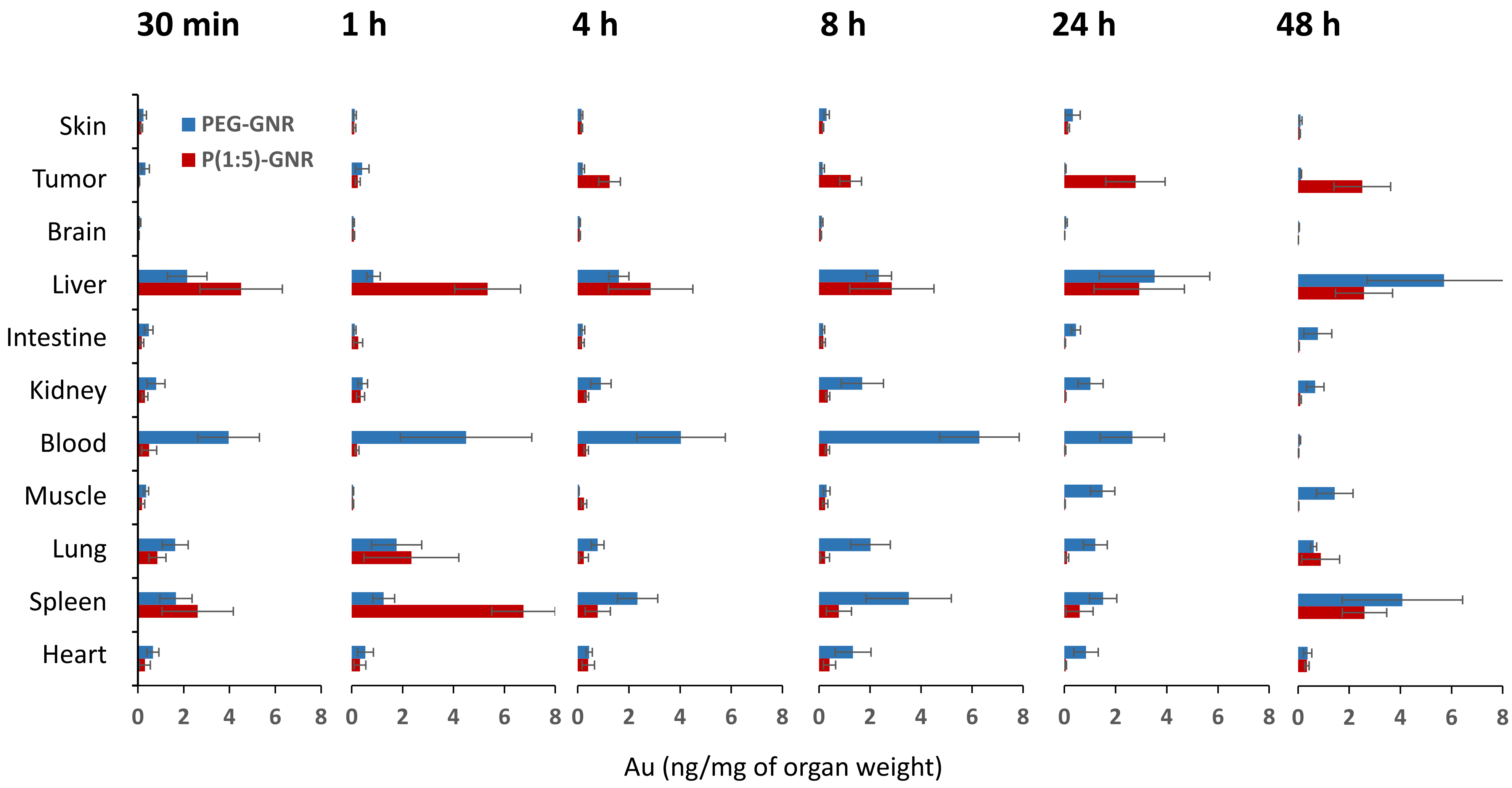
30 min
1 h
4 h
8 h
24 h
48 h
PEG-GNR
P(1:5)-GNR
Skin
Tumor
Brain
Liver
Intestine
Kidney
Blood
Muscle
Lung
Spleen
Heart
0 2 4 6 8
Au (ng/mg of organ weight)

Figure S2.

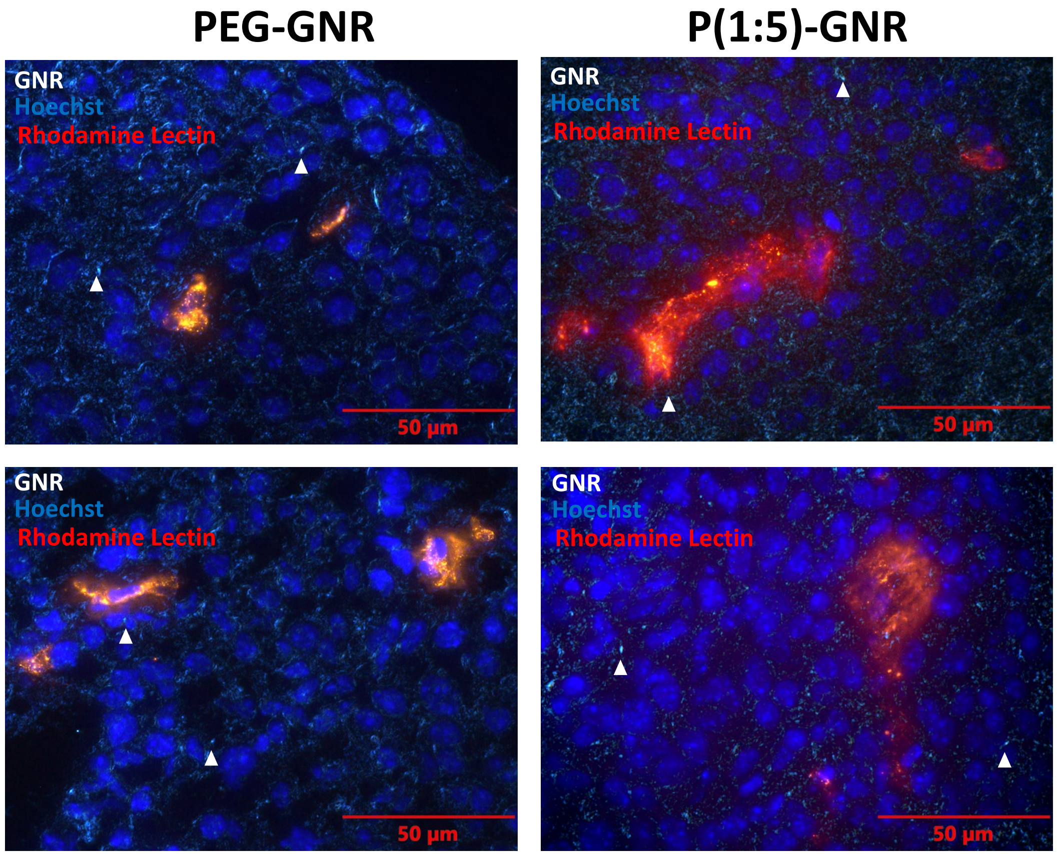
PEG-GNR
P(1:5)-GNR
GNR
Hoechst
Rhodamine Lectin
50 µm
GNR
Hoechst
Rhodamine Lectin
50 µm
GNR
Hoechst
Rhodamine Lectin
50 µm
GNR
Hoechst
Rhodamine Lectin
50 µm

Figure S3.
